# A clinical survey of the output intensity of 200 light curing units in dental offices across Maharashtra

**DOI:** 10.4103/0972-0707.57633

**Published:** 2009

**Authors:** Vivek Hegde, Sameer Jadhav, Gayatri B Aher

**Affiliations:** Department of Conservative Dentistry and Endodontics, M.A. Rangoonwala College of Dental Sciences and Research Centre, Azam Campus, Pune, Maharashtra - 411 001, India

**Keywords:** Output intensity, light-emitting diode radiometer, light curing units

## Abstract

**Aim:**

The purpose of this study is to examine the intensity of light curing units and factors affecting it in dental offices.

**Materials and Methods:**

The output intensity of 200 light curing units in dental offices across Maharashtra were examined. The collection of related information (thenumber of months of use of curing unit, the approximate number of times used in a day, and presence or absence of composite build-ups) and measurement of the intensity was performed by two operators. L.E.D Radiometer (Kerr) was used for measuring the output intensity. The average output intensity was divided into three categories (<200 mW/cm^2^, 200-400 mW/ cm^2^and >400 mW/cm^2^).

**Results:**

Among the 200 curing units examined, 81 were LED units and 119 were QTH units. Only 10% LED machines and 2% QTH curing units had good intensities (>400 mW/cm^ 2^).

**Conclusion:**

Most of the examined curing lights had low output intensity ranging from 200 to 400 mW/cm^2^, and most of the curing units had composite build-ups on them.

## INTRODUCTION

Most often, dentists blame the material for the failure of restoration rather than the technique or method of placement of restoration. Most of the studies on composite-resin curing stress the importance of sufficient output intensity of curing lights. Undesirable clinical performance and early failures of composite restorations as a result of inadequate polymerization have been reported.[1]This also includes discoloration, water absorption, and decreased hardness. Although issues such as method of placement of composite (bulk vs. incremental) and methods of curing (fast vs. slow) are not fully agreed upon among researchers and clinicians, the value of sufficient output intensity of curing lights to ensure the longevity of restorations and avoid undesirable clinical outcome is universally accepted. The impact of the sufficient intensity out put of curing lights in ensuring the longevity of restorations and avoiding undesirable clinical outcomes is universally accepted.[2]

Light-emitting diode (LED) lights have attracted interest and are becoming increasingly popular among dentists in comparison to laser and PAC lights.[3–6]The early generations of LED lights were not capable of providing adequate output (e.g., 350 mW/ cm^2^). [7]Newer generation LED lights produce output in excess of 500 mW/cm^2^. These LED lights provide more consistent outputs than the first-generation units. On average, the LED light source is expected to perform for thousands of hours in comparison to the 30-hour to 50-hour performance of quartz-tungsten-halogen (QTH) bulbs.[8] However, this long-term performance should not exempt LED lights from routine in-office evaluation.[8] Degradation of bulbs and loss of reflectors caused by heat from the filament, which are commonly seen in QTH lights, does not apply to LED lights that use gallium nitrate semiconductors as the source of luminescence.[9]

**Aim**

To examine the intensity of curing units and related factors in dental offices.

## MATERIALS AND METHODS

### Materials

L.E.D Radiometer (Kerr Manufacturing Products) was used in this study. This L.E.D Radiometer has calibrations for intensity measurement from 0 to 2000 mW/cm^ 2^. It has a detector at the centre and filter placed inside that helps in recording the intensity. The maximum and minimum diameter of detector the is 15 mm and 7 mm respectively; hence, the curing lights with tip diameter ranging from 7 mm or more can be examined by this radiometer.

### Methods

Two hundred light curing units in dental offices were examined for their output intensity. Collection of related information and measurement of the intensity was performed by two operators. Consent of the dentist was obtained in order to examine the light curing unit in the operatory.

The tip of the unit was cleaned before examination,. When a quartz-tungsten-halogen (QTH) unit was examined for the output intensity, the unit was activated for three consecutive 60-s intervals interrupted by 1 s of off time to reduce the cool bulb variable. When a light-emitting diode (LED)unit was examined, three readings were taken, and the average values were obtained. The curing units were divided into two categories: Halogen and LED. The number of years in use, the approximate no of times used in a day were asked and recorded for each curing light. Other information included bulb/ battery replacement frequency, presence or absence of composite build-ups.

The output intensity (mW/cm^2^) of all the examined lights were categorized into three groups:

1. <200 mW/cm^2^
2. 200-400 mW/cm^2^
3. >400 mW/cm^2^

The results were tabulated and statistically analyzed. (The statistical software used was SPSS version 11.5 for Windows).

## RESULTS

**Figure 1:**
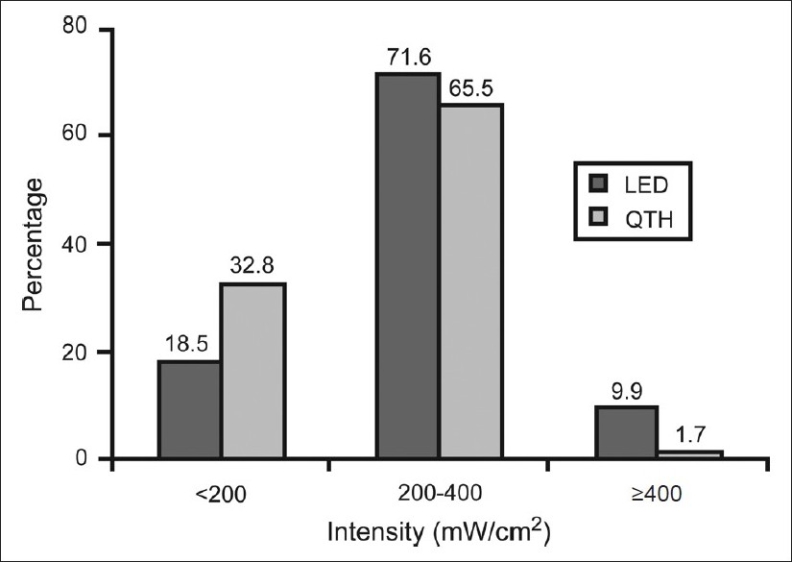
Proportion of faulty machines in use

Among the 200 curing units examined, 81 were LED units and 119 were QTH units and most of the units examined by both the operators had intensities ranging between 200 mW/cm^2^and 400 mW/cm^2^. It was observed that the battery/bulb was not changed even once in the 58/81 LED units (71.6%) and 78/119 QTH units (65%) [Table 1]. 39 (67.2%) L.E.D units and 57 QTH units (67.6%) were used for more than 36 months, and they had less output intensity compared to others [Table 2]. It was observed that the QTH units in which the bulb was changed once or twice, the recorded intensity was higher than to those in which the bulbs were not changed at all [Table 3]. There was very less difference between the readings recorded by operator 1 and operator 2 [Tables 4 and 5]. 90% of the LED units and 98% of the QTH units were found to be faulty [Figure 1].

## DISCUSSION

There are a number of factors that have a direct effect on the power intensity measured by radiometers. These include the size of the curing tip, composite resin build-up on the curing tip, the temperature of the bulb in QTH units, the orientation position of the curing tip to the radiometer, voltage regulation,the reliability of the radiometer itself, and the handling of the light curing unit.[8,10,11,17,19]

New LED radiometers have also been introduced for measuring the output intensity of LED lights. Some differences exist between LED and QTH radiometers.[11,14,18]Although both rely on a detector and a filter to measure the correct limited spectrum, they are somehow different in the way they are calibrated.[11,14,18]In a recent study, Roberts and colleagues reported that the 2 radiometers (LED and QTH) may be used interchangeably for measuring the irradiance of QTH and LED lights.[11]They also reported that the two radiometers give slightly different but correlated readings. In their study, readings from LED radiometers were slightly lower than those from QTH radiometers.[11]Irradiance values obtained from hand-held radiometers (LED or QTH) were significantly different from those obtained from a laboratory-grade power meter.[11]In general, output intensity measured using handheld radiometers are relative and not absolute values.[8,11,15]

Few machines did shut off after cyclic curing, which implies that the unit was heated up. This may delay the clinical procedure and hence warrant another stand by machine. A rise in temperature of pulp may be observed because of lower curing cycles (a cause attributed to the light pathway and intensity).

Studies have shown that the power intensity of curing lights is significantly reduced because of the presence of composite resin build-up on the curing tip.[16,17,19]Most of the curing tips examined in this study showed composite build-ups on them. It is conceivable that most, if not all, of the inadequate power densities reported in this study could be corrected or improved by eliminating build-up on the tip. However, in this study, the influence of amount of composite build-ups on the intensity of curing light was not measured; therefore, the effect of the cleaning of curing tips on the outcome of this study is unknown. a remarkable difference in intensity (by 40-50 mW/cm^ 2^) was observed when sterilization sleeves or jackets were worn on the tip of the light curing unit.

Leonard and colleagues examined the reliability of several commercial radiometers. They reported that only one built-in radiometer with a 10.5-mm tip provided an accurate measurement of the irradiance value.[8]

According to Barghi N, 30% of curing units had power densities < 199 mW/cm^ 2^and were considered inadequate for curing the composite resin. The remaining lights displayed power densities between 200 mW/cm^ 2^and 349 mW/cm^2^and were labeled to have adequate output for use with small increments of composite resin and increased curing time to ensure sufficient energy density.[12,15]

Lopes and colleagues reported that longer curing time is required to generate sufficient energy density to cure 2-mm increments of composite resin in lights with low-output intensity. An intensity of 300 mW/ cm^ 2^is low and should be compensated by a curing time of 60 s for a 2-mm increment.[13]

## CONCLUSION

Within the limitations of this study, the following conclusions could be drawn:

- Only 10% LED machines and 2% QTH machines used by practitioners were in good condition.
- Most of the curing lights examined had low output intensity ranging from 200 to 400 mW/ cm^2^.
- There is a strong association between the duration of the usage of machines and the output intensity of both the machines, i.e., more the usage, the poorer was the output intensity (*P* < 0.001 for both machines).
- Number of times of changing the battery/bulb is not associated with the intensity of LED machine; however, it is associated with the poor intensity of QTH machine.
- There is a strong agreement of similarities of results between the observers on either machines (*P* =0.000 for both machines).
- Number of times of changing the battery/bulb is not associated with the intensity of LED machine; however, it is associated with the poor intensity of QTH machine.
- There is a strong agreement of similarities of results between the observers on either machines (*P* = 0.000 for both machines).
- Most of the curing units had composite build-ups on them.

Finally to conclude, further studies can still be performed on the influence of length and diameter of the curing tip, the effect of direct current supply or battery-supported systems on the intensity of curing light, and the use of different radiometers to measure intensity for same machine.

**Table 1:** Distribution of studied parameters

	Machines	
	
	LED (n=81)	QTH (n=119)
No. of months usedҰ	36.0 (4.0–78.0)	36.0 (2.0–80.0)
No. of times battery/bulb changed		
None	58 (71.6)	78 (65.5)
Once	21 (25.9)	36 (30.3)
Twice	2 (2.5)	5 (4.2)
Intensity by operator-I		
<200 mW/cm^2^	15 (18.5)	39 (32.8)
200–400	58 (71.6)	78 (65.5)
>400	8 (9.9)	2 (1.7)
Intensity by operator-II		
<200 mW/cm^2^	14 (17.3)	37 (31.1)
200-400	58 (71.6)	80 (67.2)
>400	9 (11.1)	2 (1.7)

Ұ Values are median (minimum-maximum) values are n (%)

**Table 2:** Association with duration of use (months)

Machines	No. of months used				*P* value	
		
	<36 months		>36 months		LED	QTH
				
	LED	QTH	LED	QTH		
No. of times battery changed						
None	19 (82.6)	21 (75.0)	39 (67.2)	57 (67.6)	0.326	0.306
Once	4 (17.4)	7 (25.0)	7 (25.0)	29 (31.9)		
Twice	0	0	2 (3.4)	5 (5.5)		
Intensity						
200 mW/cm^2^		1 (3.6)	15 (25.9)	38 (41.8)	0.000	0.000
200–400	15 (65.2)	25 (89.3)	43 (74.1)	53 (58.2)		
>400	8 (34.8)	2 (7.1)	0	0		

Values are n (%), *P* values by Chi-square test

**Table 3:** Association with number of times the battery or bulb is changed

Machine	No. of times battery changed				*P* value	
		
	None		Once or twice		LED	QTH
				
	LED	QTH	LED	QTH		
Intensity						
<200 mW/cm^2^	10 (17.2)	31 (39.7)	5 (21.7)	8 (19.5)	0.709	0.048
200–400	43 (74.1)	48 (59.0)	32 (78.0)	53 (58.2)		
>400	5 (8.6)	1 (1.3)	3 (13.0)	1 (2.4)		

**Table 4:** Agreement between two independent operators (intensity-light-emitting diode)

		Operator-II		
		
	Intensity mW/cm^2^	<200	200–400	>400
Operator-I	<200	14 (93.3)	1 (6.7)	0
	200–400	0	57(98.3)	1(1.7)
	>400	0	0	8 (100.0)

Values are n (%), *P* value 5 0.000 by Chi-square test, suggesting strong agreement between two independent operators

**Table 5:** Agreement between two independent operators (intensity - quartz-tungsten-halogen)

		Operator-II		
		
	Intensity mW/cm^2^	<200	200–400	>400
Operator-I	<200	37 (94.9)	2 (5.1)	0
	200–400	0	78 (100.0)	0
	>400	0	0	2 (100.0)

Values are n(%), P value = 0.000 by Chi-square test, suggesting strong agreement between two independent operators

## References

[1] Leung RI, Fan PL, Johnston WM (1983). Post-irradiation polymerization of visible light activated composite resin. J Dent Res.

[2] Parson GJ, Longman CM (1989). Water sorption and solubility of resin-based materials following inadequate polymerization by a visible light-curing system. J Oral Rehabil.

[3] Asmussen E, Peutzfeldt A (2002). Light-emitting diode curing: Influence on selected properties of resin composites. Quintessence Int.

[4] Barghi N, McAlister EH (2003). LED and halogen lights: Effect of ceramic thickness and shade on curing luting resin. Compend Contin Educ Dent.

[5] Mills RW, Uhl A, Blackwell GB, Jandt KD (2002). High power light emitting diode (LED) arrays versus halogen light polymerization of oral biomaterials: Barcol hardness, compressive strength and radiometric properties. Biomaterials.

[6] Stahl F, Ashworth SH, Jandt KD, Mills RW (2000). Light-emitting diode (LED) polymerization of dental composites: Flexural properties and polymerization potential. Biomaterials.

[7] Hammesfahr PD, O'Connor MT, Wang X (2002). Light-curing technology: Past, present, and future. Compend Cont Educ Dent.

[8] Leonard DL, Charlton DG, Hilton TJ (1999). Effect of curing-tip diameter on the accuracy of dental radiometers. Oper Dent.

[9] Burgess JO, Walker RS, Porche CJ, Rappold AJ (2002). Light curing: Anupdate. Compend Contin Educ Dent.

[10] Davidson CL, De gee AJ (2000). Light-curing units, polymerization, and clinical implications. J Adhes Dent.

[11] Roberts HW, Vandewalle KS, Berzins DW, Charlton DG (2006). Accuracy of LED and halogen radiometers using different light sources. J Esthet Restor Dent.

[12] Barghi N, Berry T, Hatton C (1994). Evaluating intensity output of curing lights in private dental offices. J Am Dent Assoc.

[13] Lopes GC, Vieira LC, Araujo E (2004). Direct composite resin restorations: A review of some clinical procedures to achieve predictable results in posterior teeth. J Esthet Restor Dent.

[14] Fan PL, Wozniak WT, Reyes WD, Stanford JW (1987). Irradiance of visible light-curing units and voltage variation effects. J Am Dent Assoc.

[15] Hasegawa T, Itoh K, Yukitani W, Wakumoto S, Hisamitsu H (2001). Effects of soft-start irradiation on the depth of cure and marginal adaptation of dentin. Oper Dent.

[16] Duke ES (2001). Light-emitting diodes in composite resin photopoly merization. Compend Contin Educ Dent.

[17] Feilzer AJ, Dooren LH, De gee AJ, Davidson CL (1995). Influence of light intensity on polymerization shrinkage and integrity of restoration-cavity interface. Eur J Oral Sci.

[18] Rueggeberg FA, Caughman WF, Curtis Jr JW (1994). Effect of light intensity and exposure duration on cure of resin composite. Oper Dent.

[19] Rueggeberg FA, Caughman WF, Curtis JW, Davis HC (1993). Factors affecting cure at depths within light-activated resin composites. Am J Dent.

